# What is constant in a time of change?

**DOI:** 10.3325/cmj.2011.52.593

**Published:** 2011-10

**Authors:** Srećko Gajović

Nowadays, not only medicine is under the pressure of rapid change and transformation. This is characteristic for every discipline; moreover, the complexity of the changes that are taking place has had the effect on every sphere of life, even the individual sphere. The results are not always positive and the consequences could be harsh and out of control of the affected individuals. The global economy, global warming, global informatization, and other “global” phenomena add to the uncontrollable spin of events generated at the local level. These global-local combinations result in important novel opportunities but also in novel risks for everyone. Although it can seem paradoxical in the time of rapid changes, a possible answer to the increasing challenges could be a retreat toward basic questions. Which basic issues add to the quality of our life? Which basic values are worth preserving? On what foundations can we build the continuous adaptation necessary to cope with the complex changes? I believe that increasing challenges around us should make everybody question the validity of the very basic postulates of our lives. If we adopt this standpoint, the results of the Cover Page study by Radin, Džakula, and Benković in this issue of the *Croatian Medical Journal* are not at all surprising (1). The study used the data collected through the telephone interviewing in order to test the pre-election public opinion in the last three Croatian elections. When asked about the most important election issue, the voters, surprisingly indeed, repeatedly chose – health! These results are particularly valuable in the eve of the 2011 Croatian elections. The identification of health as a priority challenges the domination of other concurrent issues related to the immediate situation. Health was chosen as a priority in Croatia, a typical country in the process of transition and accession to the European Union, where the changes should be even more accelerated and aggressive. It may be indeed surprising for the political establishment facing the upcoming election that through all this time of changes in Croatia, in 2005, 2007, and 2009, health stubbornly remained among the two most important election issues for the voters. The importance of health documented in this study reveals not only voters’ standpoints, but also an important constant in the change. The health issue certainly needs to be addressed by strategies presented to the voters and implemented after the election.

The *Croatian Medical Journal* is also challenged to cope with the time of change, hence it should ask itself what its basic postulates are. Again we believe we should hold on to the basic principles of biomedical research to provide a common ground for the increasing number of topics offered to a general medical journal. Therefore, we will continue assessing the manuscripts not on the basis of whether they are a priority in science, or whether they represent a hot topic everybody would like to publish, but on the basis of their scientific merit, quality of the study design, and well documented results. We strongly believe that only the quality of presented research is the fundamental for the further development of the *Croatian Medical Journal*, and this is the value with which we face the future of changes. In medicine in particular, the decisions every doctor has to make should be based on the facts, the facts which stem from the research data. Assuring the reliability of these data is the mission of every medical journal.

Behind the curtain of this struggle for scientific quality, with authors and referees acting as the major stakeholders, the *Croatian Medical Journal* has faced another type of change, the change of the Editorial Board and the Editor-in-Chief. Again, the change is a motive for revealing the fundamentals and reaffirming the *Croatian Medical Journal’s* basic values. These are incorporated in the author-friendly policy of turning valuable research into high-quality manuscripts. Striving for excellence is our constant, which serves to achieve the journal’s progress. If the realization of this approach is successful, the *Croatian Medical Journal* will prosper. In return, this would grant the people taking care about the journal the deserved satisfaction for their efforts.
